# Application of B-ultrasound for localization and its impact on first-attempt success rates of nasogastric tube insertion in critically ill patients

**DOI:** 10.1097/MD.0000000000036452

**Published:** 2023-12-15

**Authors:** Lijuan Gao, Xiajuan Luo, Huijie Deng, Ni Shi, Xiaohua Wen

**Affiliations:** a Department of Critical Care Medicine, Zhujiang Hospital, Southern Medical University, Guangzhou, Guangdong, China.

**Keywords:** blind interpolation, B-ultrasound positioning, critically ill patients, nasointestinal tube implantation

## Abstract

The objective of this study is to explore the application effect of B-ultrasound positioning in assisting nasointestinal tube implantation in critically ill patients. This study is a retrospective study. In this study, 90 cases of severe patients with nasointestinal tube implantation were included. According to the different ways of nasointestinal tube insertion received by patients, 61 patients with conventional blind insertion methods were included in the blind insertion group, and 29 patients with conventional methods and B-ultrasound assisted positioning were included in the B-ultrasound positioning group. The general clinical data, success rate of catheterization, catheterization time, pyloric passage rate, and target nutritional value time of the 2 groups were compared. The changes of the 2 groups after catheterization were compared by SOFA and APACHE II. The contents of albumin and lymphocyte count were compared between the 2 groups before and after catheterization. The time of target nutritional value of the patients of the B-ultrasound positioning group was markedly decreased comparing with the patients of the blind insertion group. The index of catheterization time of the patients between the blind insertion group and B-ultrasound positioning group had no obvious contrast. The APACHE II score and SOFA score of the patients of the B-ultrasound positioning group were obviously lower than the blind insertion group. The contents of lymphocyte count of the patients of the B-ultrasound positioning group were markedly increased comparing with the patients of the blind insertion group after catheterization, but the contents of albumin content had no obvious change. The scores of respiratory system, circulatory system, nervous system, and urinary system in the B-ultrasound positioning group were significantly higher than those in the blind insertion group, while the COPT scores were significantly lower than those in the blind insertion group. B-ultrasound assisted nasointestinal tube implantation is well tolerated in critically ill patients, and can effectively ameliorate the nutritional status and of the ill patients.

## 1. Introduction

The treatment of clinically critical patients is predominantly conducted in intensive care units (ICUs). Patients in ICUs exhibit high metabolic rates and rapid decomposition within the body. These ill patients often find themselves in a state of negative nitrogen balance and increased secretion of catabolic hormones, resulting in varying degrees of malnutrition. Such malnutrition can adversely affect rehabilitation outcomes, in addition to causing a range of complications that could potentially endanger the lives of patients.^[1–3]^

Early enteral nutrition has been proven to ameliorate the immunosuppressive state of critically ill patients, as well as rectify internal environment disorders such as negative nitrogen balance during acute stress periods. Furthermore, it can stimulate intestinal peristalsis, prevent displacement of intestinal flora, and circumvent enterogenous infections. Collectively, these benefits contribute to improved patient conditions and prognoses, earning early enteral nutrition unanimous recommendations in international guidelines.^[4,5]^ Post-pyloric parenteral nutrition is advantageous in that it is well-tolerated, reduces the likelihood of reflux aspiration, achieves high utilization rates of nutrient absorption, provides sufficient nutrition in a short timeframe, and is associated with fewer complications.^[1]^ Presently, all international nutrition guidelines advocate for the initiation of early enteral nutrition in critically ill patients, with some even suggesting that enteral nutrition should commence within 24 to 48 hours of ICU admission to optimize patient outcomes.^[6,7]^ During the acute phase, most critically ill patients experience immunosuppression, and internal environment disorders such as negative nitrogen balance may also occur. Early enteral nutrition can foster smooth transitions for critically ill patients through these acute phases by stimulating intestinal peristalsis, preventing displacement of intestinal flora, and avoiding enterogenous infections.^[8,9]^ With the growing recognition of the value of post-pyloric enteral nutrition in critically ill patients, establishing an appropriate enteral nutrition pathway is vital to ensuring the commencement of early enteral nutrition in these patients.^[10]^

Currently, the preferred method of maintaining the nutritional status of ICU patients is enteral nutrition. This involves the delivery of sufficient nutrients to the patient’s body via nasal feeding or oral feeding, thereby facilitating the recovery of intestinal peristalsis, regulating immune function, and providing the necessary cellular metabolic capabilities.^[11–13]^ To expedite patient progress towards target nutritional values and minimize adverse reactions, a safe, convenient, and effective method for inserting nasointestinal tubes is required. With the advent of medical science and technology, B-ultrasound has increasingly been applied to nasointestinal tube indwelling, yielding remarkable results. However, to date, there has been a paucity of research exploring the application effect of B-ultrasound positioning in assisting the placement of nasal and intestinal tubes in severely ill patients. This study investigates the application effect of B-ultrasound-assisted nasointestinal tube placement in critically ill patients, comparing blind insertion and B-ultrasound-assisted placement methods.

## 2. Methods

### 2.1. Patients

This study is a retrospective analysis that received approval from the Ethics Committee of Zhujiang Hospital. A total of 90 critically ill patients who underwent nasointestinal tube implantation at the hospital from January 2022 to February 2023 were included in the study. The cohort consisted of 68 male and 22 female patients, with a mean Glasgow Coma Score (GCS) of 8.07 ± 3.02. Patients in the B-ultrasound positioning group had their nasointestinal tubes placed with the assistance of B-ultrasound positioning.

### 2.2. Inclusion Criteria

(1) Patients met the admission criteria for the ICU based on clinical examination. (2) Enteral nutrition support was required for patients for a duration of >1 month. (3) The patient’s age must be between 18 and 70 years old, with no serious cardiovascular diseases or other malignant tumors. (4) The patient’s information and data relevant to this study must be complete and available.

### 2.3. Exclusion criteria

(1) The patient had recently undergone major surgery or chemotherapy. (2) The patient had primary digestive system disease. (3) The patient had severe liver, kidney, or immune system diseases. (4) The patient had esophageal injuries or throat disease. (5) The patient suffered from severe mental illness. (6) The patient refused to participate in the study or withdrew partway through the study.

### 2.4. Catheterization methods

#### 2.4.1. Blind insertion group.

Patients in this group underwent blind insertion with a Coolpad 140 guidewire; 3 minutes before the procedure, patients were injected intravenously with 10 mg of metoclopramide, and the head of the bed was raised to 40°. The patients were intubated using a blind insertion method in a semi-reclining position. The nasointestinal tube was then inserted into the gastric fundus and auscultated at the gastric antrum after being placed at a depth of 60 cm. Gas was injected to confirm that the tube had entered the gastric cavity. The tube was then advanced 20 to 30 cm further (auscultation was performed at the duodenal bulb, horizontal part, and left lower abdomen at 70 cm, 80 cm, and 90 cm, respectively). The guidewire was removed, and the tube was fixed in place. The direction of the nasointestinal tube and the position of the tip of the nutrition tube were verified by a plain film of the bedside abdomen on the day of tube placement.

#### 2.4.2. B-ultrasound positioning method.

**Figure 1.**
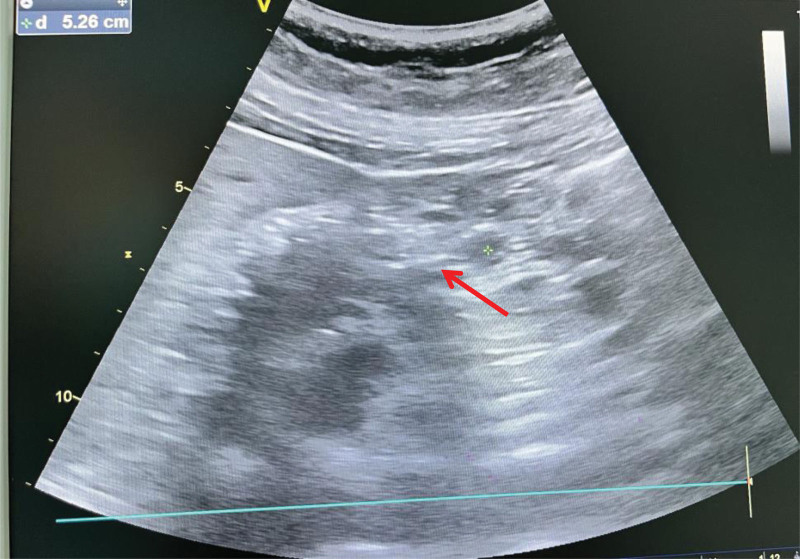
The “double track” sound image in the B-ultrasound pyloric imaging.

Three minutes before the procedure, the patient was injected intravenously with 10 mg of metoclopramide, and the head of the bed was raised to about 40°. The patient was kept in a semi-reclining position. The Coolpad 140 guidewire was inserted into the nasal cavity to a depth of about 50 cm; 5 mL of gas was quickly injected into the nasointestinal tube, and the gastric vesicle area was auscultated to confirm that the tube was properly placed in the gastric cavity. Next, 30 mL of 0.9% sodium chloride solution was quickly injected into the stomach. Under B-ultrasound guidance (GE, 3.5 MHz) and based on pyloric imaging, the catheter was twisted and pushed into the nasointestinal tube at a speed of 2 cm per minute. If significant resistance was felt, the catheter was withdrawn 5 to 10 cm until there was no rebound feeling after reinserting the guidewire, and the process was repeated. A “double track” sound image in the B-ultrasound pyloric imaging indicated that the catheter had passed through the pyloric tube (Fig. 1). At this point, the catheter was advanced at least 20 cm further, and the guidewire was removed. A small amount of 0.9% sodium chloride solution was injected to confirm that the nasointestinal tube was unobstructed, and the catheter was properly fixed.

After the patient’s bowel sounds had recovered and hemodynamics were stable, an enteral nutrient solution was administered through the nasointestinal tube at a rate of 20 to 25 mL per hour on the same day. If the patient did not experience diarrhea, abdominal distension, or aspiration, the rate was increased to the target nutrient value on the next day.

### 2.5. Observation index

The general clinical data of the 2 groups were recorded, including the success rate of catheterization and pyloric passage rate, as well as the time required for catheterization and the time taken to reach the target nutritional value. The APACHE II score and SOFA score were utilized to compare the changes in the 2 groups post-catheterization. Additionally, the levels of albumin and lymphocyte count before and after catheterization were compared between the 2 groups. *Clinical Symptom Score*: The patient’s circulatory, digestive, respiratory, urinary, and nervous systems were evaluated using a systematic symptom evaluation scale, with a total score of 30 points. A higher score indicates a more significant improvement in symptoms. The COPT scale was employed to assess pain, including facial expression, movement, muscle tension, and phonation/compliance with mechanical ventilation, with each item scored from 0 to 2 points. The total score ranges from 0 to 8 points, with 0 indicating no pain and 8 indicating the most severe pain.

### 2.6. Statistical analysis

In this study, SPSS 26.0 software was used for data analysis. The measurement data were expressed in the form of mean ± variance, and the data between the 2 groups are compared by *t* test. The counting data were expressed in the form of cases (percentage), and the data between the 2 groups were compared by chi-square test.

## 3. Results

### 3.1. Comparison of clinical data of the patients in the 2 groups

The terms of general clinical data of age, sex, BMI, APACHE II score, ISS score, SOFA score, diagnosis results, and GCS score in the blind insertion group and the B-ultrasound positioning group had no obvious difference (Table 1).

**Table 1:** Comparison of general clinical data between the 2 groups.

Clinical data	Blind insertion group (n = 61)	B-ultrasound positioning group (n = 29)	*X*^2^/*t*	*P* value
Age (yr)	53.64 ± 12.75	51.08 ± 11.32	0.922	.359
Gender (male, %)	47 (70.00%)	21 (60.00%)	0.229	.633
BMI	23.67 ± 4.85	23.93 ± 5.74	0.224	.823
APACHE II score	29.60 ± 6.24	30.40 ± 5.84	0.580	.563
SOFA score	12.10 ± 4.69	11.50 ± 3.45	0.614	.541
Diagnosis			4.557	.102
Head trauma (%)	14 (30.00%)	12 (20.00%)		
Respiratory diseases (%)	12 (10.00%)	7 (30.00%)		
Other (%)	35 (20.00%)	10 (40.00%)		
GCS	8.90 ± 4.09	9.30 ± 5.01	0.403	.688

GCS = Glasgow Coma Score.

### 3.2. Comparison of clinical indexes of the patients in the 2 groups

**Figure 2.**
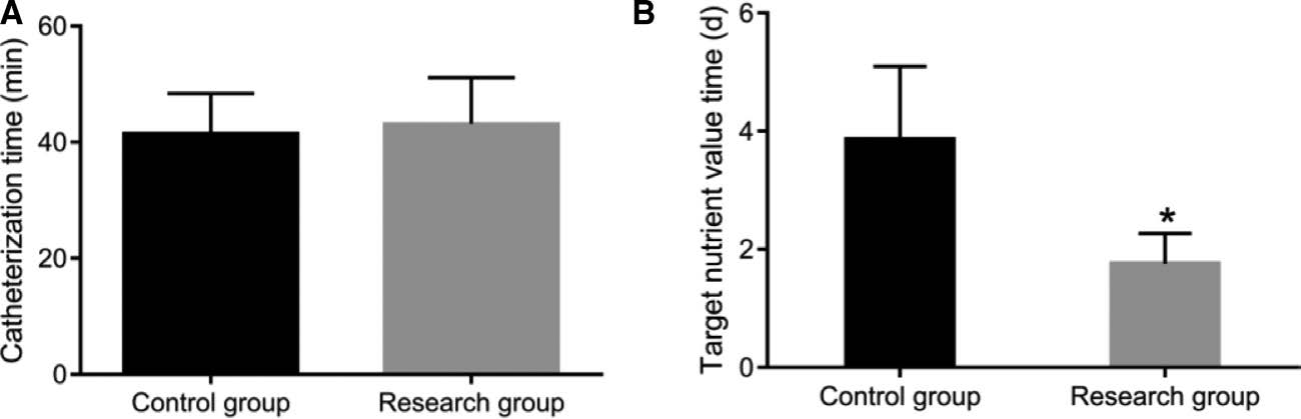
Comparison of clinical indexes of patients in the blind insertion group and the B-ultrasound positioning group: catheterization time (A) and target nutritional value time (B). *P < .05.

After nasointestinal tube implantation, target nutrition value time (Fig. 2B) and insertion tube (Fig. 2A) of patients in the B-ultrasound positioning group and blind insertion group were compared. We found that the implantation success rate of patients in the B ultrasound positioning group was significantly higher than that in the blind group (Table 2, *P* < .05), and the time of nasoenteric tube implantation and the time to reach the target nutritional value was lower than that in the blind group. The above results indicate that compared with the blind insertion group, the placement of nasal and intestinal tubes assisted by B-ultrasound can significantly shorten the time to reach the target nutritional value and improve the success rate of tube placement.

**Table 2:** The success of nasoenteric tube implantation for one time between 2 groups.

Group	The success of nasoenteric tube implantation for one time	Success rate
Control group	50	82.00%
Research group	28	96.55%
Statistical value	*P* < .05	

### 3.3. Evaluation of patients’ condition after nasointestinal tube implantation in the 2 groups

In order to explore the influence of patients in the blind insertion group and the B-ultrasound positioning group on their condition after nasointestinal tube implantation, the APACHE II and SOFA score were evaluated of the condition of patients in the 2 groups. The results showed that the APACHE II and SOFA score of patients in the B-ultrasound positioning group were lower than the blind insertion group (Table 3). The above results showed that B-ultrasound assisted nasointestinal tube implantation can significantly improve the patient’s condition.

**Table 3:** Comparison of APACHE II and SOFA score after nasointestinal tube implantation.

Group	n	APACHE II score	SOFA score
Blind insertion group	61	28.50 ± 6.45	11.50 ± 3.45
B-ultrasound positioning group	29	18.80 ± 5.96	7.60 ± 2.27
*t*		3.493	2.986
*P*		.003	.008

### 3.4. Comparison of clinical symptom scores of patients

**Figure 3.**
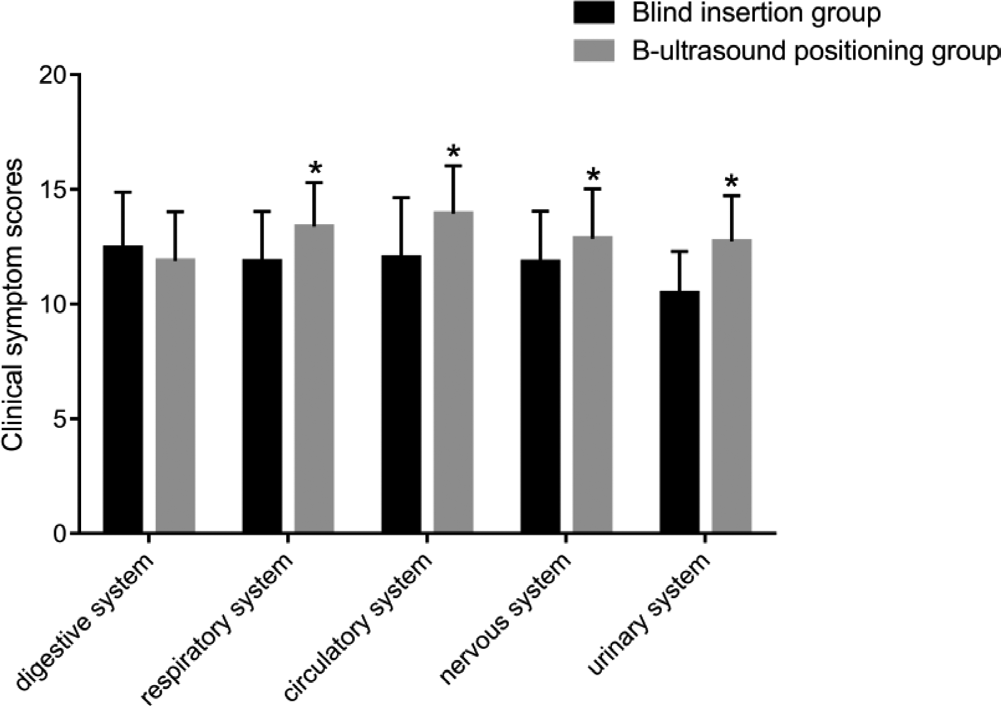
Comparison of clinical symptom scores between blind insertion group and B-ultrasound positioning group. *P < .05.

Comparing the clinical symptom scores of patients in blind insertion group and B-ultrasonic positioning group (Fig. 3). The results showed that the scores of respiratory system, circulatory system, nervous system, and urinary system in the B ultrasonic positioning group were significantly higher than those in the blind insertion group. There was no significant difference in digestive system scores between blind insertion group and B-ultrasonic positioning group.

### 3.5. Comparison of pain scores of patients

**Figure 4.**
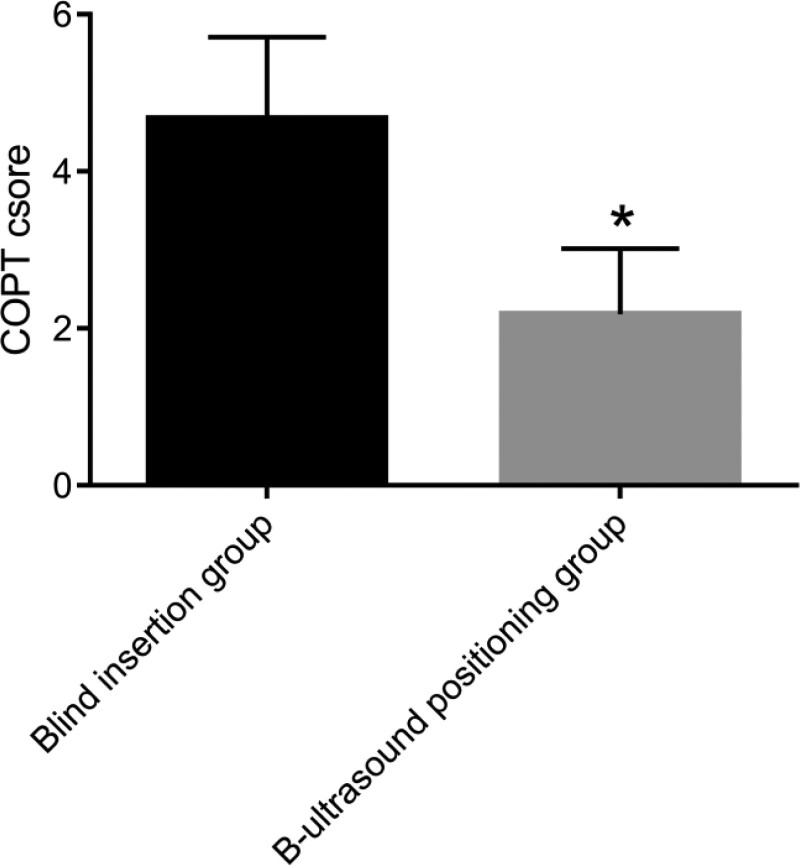
Comparison of COPT scores between blind insertion group and B-ultrasound positioning group. *P < .05.

The pain of patients after nasointestinal tube insertion was compared (Fig. 4). The COPT scale showed that the score of patients in the B-ultrasonic positioning group was significantly lower than that in the blind insertion group. The above results indicate that the placement of nasointestinal tube assisted by B-ultrasound localization has a very significant effect on relieving the pain of patients.

### 3.6. Comparison of nutritional indexes between the 2 groups

We compared the albumin content and lymphocyte count of patients in the B-ultrasound positioning group and the blind insertion group before and after catheterization. Our findings showed that the lymphocyte count of patients in the B-ultrasound positioning group post-catheterization was higher than that in the blind insertion group (Table 4).

**Table 4:** Comparison of nutritional indexes before and after nasointestinal tube implantation.

		Albumin (g/L)	Lymphocyte
Before catheterization	Blind insertion group	35.48 ± 5.32	3.90 ± 1.29
	B-ultrasound positioning group	34.64 ± 5.52	4.00 ± 1.20
* t*		0.347	0.180
* P*		.733	.860
After catheterization	Blind insertion group	34.61 ± 4.75	3.10 ± 1.09
	B-ultrasound positioning group	33.96 ± 4.53	1.90 ± 0.69
* t*		0.313	2.942
* P*		.758	.009

## 4. Discussion

When critically ill patients are in a relatively stable condition, it is necessary to initiate gastrointestinal nutrition promptly. Currently, enteral nutrition is considered the preferred choice, typically administered through a nasointestinal tube. Therefore, the accurate placement of the nasointestinal tube is crucial to avoid related complications.^[14,15]^ Enteral nutrition through a nasoenteral tube holds significant clinical importance for critically ill patients, as it can effectively reduce the likelihood of aspiration pneumonia resulting from gastrointestinal reflux.^[16]^

Conventional methods for nasointestinal tube placement include endoscopic-assisted tube placement, X-ray fluoroscopy-assisted tube placement, and blind insertion.^[12,17,18]^ Endoscopic-assisted catheterization currently boasts one of the highest success rates, but its invasiveness, complex procedure, time-consuming nature, high cost, and associated risks limit its usage and promotion for nasointestinal tube implantation.^[19–21]^ Although abdominal X-ray film is considered the “gold standard,” ionizing radiation exposure during X-rays increases the risk of radiation injury to both doctors and patients. Other methods often lack sensitivity and accuracy due to various influencing factors.^[22–24]^ Although innovations and improvements have been made in positioning methodology, such as the injection of methylene blue, gastric electrode monitoring, and magnetic navigation, these methods are subject to strong subjectivity and can be affected by gastrointestinal function.^[25–27]^ While the blind insertion method does not require specialized instruments, the human body’s complex anatomical structure lowers the success rate of catheterization, lengthens the time required for the tube to pass through the pylorus, and necessitates multiple X-ray exams to determine the catheter tip’s position.^[28–30]^

In this study, ultrasound-guided nasointestinal catheterization was utilized, combining ultrasonic positioning during catheterization to monitor the catheter tip’s position in real-time. This improved the pyloric passing rate and overall success rate of catheterization. Given the critical condition of ICU patients and their pain and intolerance towards nasointestinal tube implantation, propofol was used for sedation, sufentanil for auxiliary analgesia, and metoclopramide administered orally to prevent nausea and vomiting caused by tube implantation.^[31,32][33]^ The results suggested that B-ultrasound-assisted nasointestinal tube insertion was superior to blind insertion regarding the time to reach the target nutritional value. Furthermore, B-ultrasound-guided intragastric water injection for nasointestinal tube placement could potentially shorten the time to reach target nutritional value. Rapidly injecting a suitable amount of 0.9% sodium chloride solution into the stomach via the catheter can expose the patient’s pyloric tube. The specific position of the metal guide wire of the gastric nutrition tube can be precisely located under B-ultrasound guidance, thus avoiding the blindness associated with catheterization. Additionally, our findings indicated that B-ultrasound-assisted localization could more effectively improve the patient’s condition and nutritional status, enhance their immunity, and subsequently improve their prognosis.

However, this study is not without limitations. The small sample size necessitates the collection of more data to further investigate the role of B-ultrasound localization in the placement of nasointestinal tubes in critically ill patients. Despite this, the accuracy of the results remains unaffected. Our future research endeavors will aim to expand the sample size, conduct multi-center studies where feasible, and incorporate follow-ups to explore more effective methods of enteral nutrition.

## 5. Conclusion

In conclusion, B-ultrasound positioning assisted nasointestinal tube implantation has shown promising results in critically ill patients. This method offers a simple, safe, and highly successful approach with good patient tolerance. It is a valuable addition to the current methods of nasointestinal tube implantation and is worth considering for wider clinical adoption.

## Author contributions

**Conceptualization:** Lijuan Gao, Xiajuan Luo, Huijie Deng, Xiaohua Wen.

**Data curation:** Lijuan Gao, Ni Shi.

**Formal analysis:** Lijuan Gao, Huijie Deng.

**Investigation:** Lijuan Gao, Xiajuan Luo, Huijie Deng, Ni Shi, Xiaohua Wen.

**Methodology:** Lijuan Gao, Xiajuan Luo, Huijie Deng, Ni Shi, Xiaohua Wen.

**Supervision:** Xiajuan Luo, Ni Shi, Xiaohua Wen.

**Visualization:** Ni Shi, Xiaohua Wen.

**Writing – original draft:** Lijuan Gao.

**Writing – review & editing:** Lijuan Gao, Xiajuan Luo.
